# Variable Region Sequences Influence 16S rRNA Performance

**DOI:** 10.1128/spectrum.01252-23

**Published:** 2023-05-22

**Authors:** Nikhil Bose, Sean D. Moore

**Affiliations:** a Burnett School of Biomedical Sciences, College of Medicine, University of Central Florida, Orlando, Florida, USA; Northwestern University

**Keywords:** 16S rRNA, variable region, single nucleotide polymorphism, relative entropy, ribosome quality, taxonomy

## Abstract

16S rRNA gene sequences are commonly analyzed for taxonomic and phylogenetic studies because they contain variable regions that can help distinguish different genera. However, intra-genus distinction using variable region homology is often impossible due to the high overall sequence identities among closely related species, even though some residues may be conserved within respective species. Using a computational method that included the allelic diversity within individual genomes, we discovered that certain Escherichia and *Shigella* species can be distinguished by a multi-allelic 16S rRNA variable region single nucleotide polymorphism (SNP). To evaluate the performance of 16S rRNAs with altered variable regions, we developed an *in vivo* system that measures the acceptance and distribution of variant 16S rRNAs into a large pool of natural versions supporting normal translation and growth. We found that 16S rRNAs containing evolutionarily disparate variable regions were underpopulated both in ribosomes and in active translation pools, even for an SNP. Overall, this study revealed that variable region sequences can substantially influence the performance of 16S rRNAs and that this biological constraint can be leveraged to justify refining taxonomic assignments of variable region sequence data.

**IMPORTANCE** This study reevaluates the notion that 16S rRNA gene variable region sequences are uninformative for intra-genus classification and that single nucleotide variations within them have no consequence to strains that bear them. We demonstrated that the performance of 16S rRNAs in Escherichia coli can be negatively impacted by sequence changes in variable regions, even for single nucleotide changes that are native to closely related Escherichia and *Shigella* species; thus, biological performance is likely constraining the evolution of variable regions in bacteria. Further, the native nucleotide variations we tested occur in all strains of their respective species and across their multiple 16S rRNA gene copies, suggesting that these species evolved beyond what would be discerned from a consensus sequence comparison. Therefore, this work also reveals that the multiple 16S rRNA gene alleles found in most bacteria can provide more informative phylogenetic and taxonomic detail than a single reference allele.

## INTRODUCTION

The small subunits of bacterial and archaeal ribosomes contain 16S rRNAs, which are required for particle assembly, ribosome formation (1–5), and for decoding mRNAs (6, 7). The *rrs* genes encoding 16S rRNAs have diverged over time, with regions encoding biochemically important residues changing infrequently and other regions exhibiting much greater sequence diversity (variable regions) (8, 9). It is commonly assumed that these variable regions play little to no role in the performance of the 16S rRNA, so their sequences can serve as 'molecular timers' that reflect evolutionary distance (10). Most interrogations of the functionalities of 16S rRNAs have focused on highly conserved regions because of the broad interest in understanding the biochemistry of protein synthesis (11–13). However, much less is known regarding the impact of sequence changes in variable regions, which are unlikely to influence the biochemistry of translation but may influence small subunit assembly or conformational dynamics, potentially constraining their evolution.

Shannon entropy calculations have been used to computationally evaluate the conservation of 16S gene residues across bacteria (14, 15), which confirmed the presence of 9 variable regions (V1 to V9). These variable regions are commonly analyzed for microbial classification, with the combined V3-V4 segment being evaluated the most because its length accommodates affordable second-generation sequencing technologies (16–21). Unfortunately, categorizing 16S rRNA variable region sequences to the species level is not regularly achieved because the few (but potentially informative) residue differences are computationally outweighed by overwhelming similarities in the remainder of the sequences (15, 22). This problem is exemplified when variable region analyses co-classify sequences as both Escherichia and *Shigella* (23–25), even though 16S gene sequences differences exist between these genera and among their respective species (26, 27).

Because evolutionary relatedness is often assigned by overall sequence similarity between 2 isolated species-representative sequences, the evolutionary information contained in sequence differences among the multiple 16S gene copies in the same organism is overlooked (23, 28–30). Applying stringent sequence classification criteria (29, 30) and incorporating more 16S rRNA sequences into databases can partially alleviate this problem (22), but potential biological impacts arising from intra-genus allelic variations makes this endeavor less effective because pressures driving nucleotide conservation among variable region alleles have not been evaluated.

Among closely related groups of bacteria, there can be conservation of certain residue identities in rRNA variable regions, which suggests variable regions may not be fully capable of unbiased drifting and that biological fitness may influence the tolerance of mutations to these regions. Here, we establish that the identity of certain residues in variable regions can be strongly indicative of a particular species, which suggests a specific variable region sequence is preferred in that organism. To evaluate this idea, we developed an *in vivo* system that allowed the performance of alternative Escherichia coli 16S rRNAs to be evaluated as they progressed through small subunit assembly and entered the translation pools, even if they contained dominant lethal mutations. We discovered that altering 'species-conserved variable' residues was detrimental to the performance of the 16S rRNA, even for small changes that can distinguish the closely related Escherichia albertii, Shigella dysenteriae, and Shigella boydii. Taken together, these computational and biological evaluations of 16S rRNA variable region polymorphisms support a model that biological performance has constrained the evolutionary drift of rRNA variable regions and that simply comparing overall sequence identities overlooks potentially informative nucleotides.

## RESULTS

### Positional relative entropy reveals strain- and species-specific residue variations.

**FIG 1:**
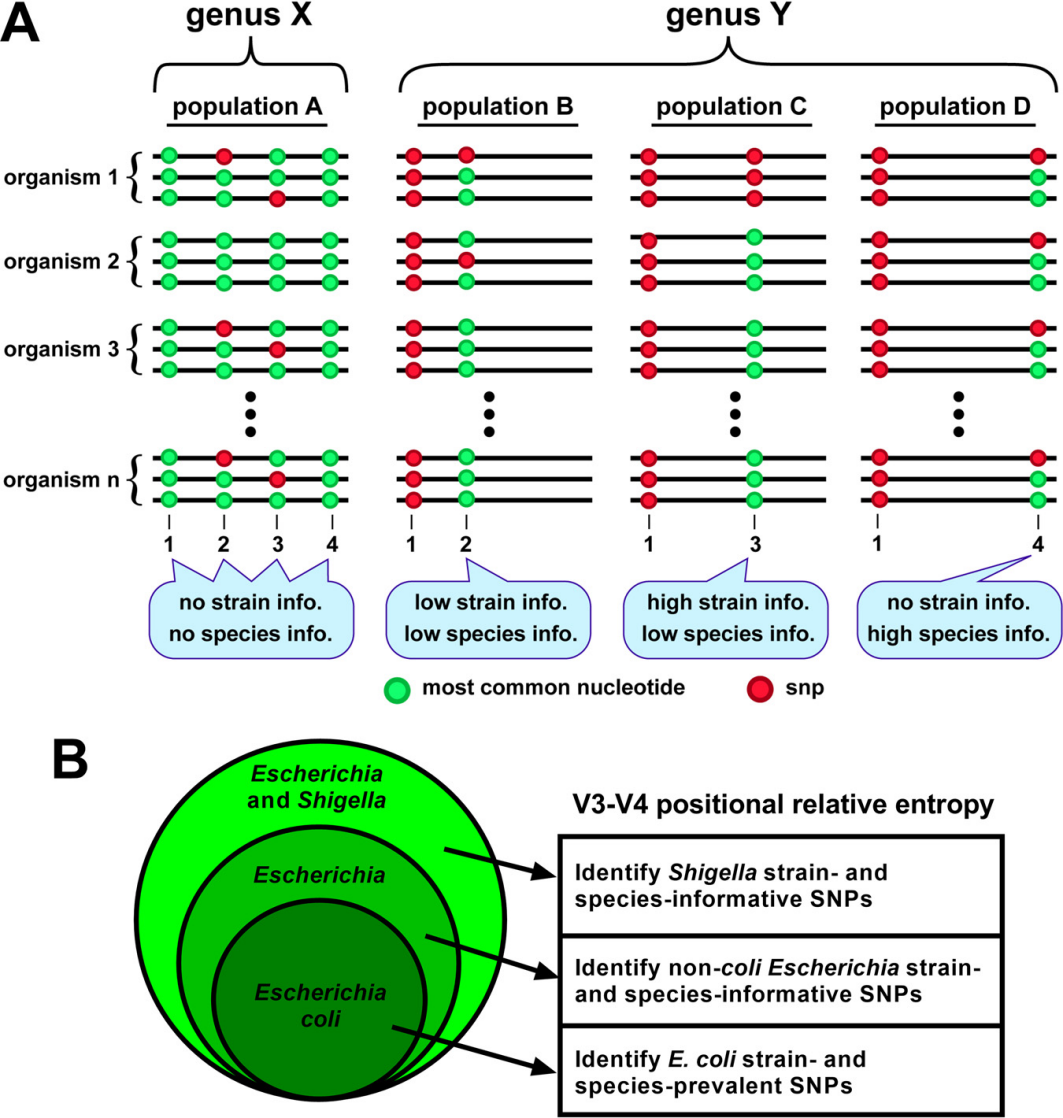
Illustration of 16S rRNA V3-V4 positional relative entropy. Relative entropy analysis can be used to identify strain- or species-specific residues among variable region sequences in a population. (A) Schematic depictions of variable regions of a multi-copy gene found in 2 hypothetical genera, X, and Y. In a sequenced cohort of genus X organisms (population A), invariant residues at positions 1 and 4 provide no sub-genus information, and SNPs observed at positions 2 and 3 are not associated with a particular species or strain, so their identities provide no information at those taxonomic levels. In genus Y, the SNP at position 1 is a strong genus indicator (relative to that of genus X). In population B, occasional SNPs at position 2 provide no information because they are observed in single alleles in strains nonspecific to a species. In population C, an SNP at position 3 is a strong strain indicator because it is present in all alleles in the strain’s genome. In population D, an occasional SNP at position 4 indicates the presence of that species but provides no strain information. (B) A Venn diagram illustrating the process of using positional relative entropy (*D*_KL_) to identify informative V3-V4 residues, starting from (i) those prevalent in a strain or multiple strains relative to the total E. coli population, then (ii) those informative of non-*coli* Escherichia strains and species relative to the total Escherichia population, and lastly (iii) those informative of *Shigella* strains and species relative to the total Escherichia and *Shigella* population.

To evaluate the capability of intra-genus variable region sequences to provide taxonomically-informative single nucleotide polymorphisms (SNP), we focused on interrogating the ~450 nucleotide 16S rRNA V3-V4 region because of its prominent use and extensive reference data sets from environmental and clinical studies (18–21). The Escherichia and *Shigella* V3-V4 regions typically have only 1 to 4 residue differences among them, which have thus far been of no utility for species classifications based on homology. Escherichia and *Shigella* strains typically have 7 copies of the *rrs* gene, each of which may have varying residues. To gauge the evolutionary change observed in a strain relative to the population, we computed a relative entropy (Kullback-Leibler divergence [*D*_KL_]) (31) to compare the residue frequency observed at a V3-V4 position across alleles within a strain to the frequency at that position for the overall population (Fig. 1A). Therefore, a residue variation in a single allele of only 1 organism of the population indicates less evolution, yields a low *D*_KL_ value, and is less strain-informative. The same residue variation in all 7 alleles of only 1 strain in the population indicates greater evolution, yields a high *D*_KL_ value, and is more strain-informative. By taking the cumulative sum of *D*_KL_ (c*D*_KL_) at a position across strains in a population, we determined if the variation was prevalent in many strains and potentially species-informative.

We obtained V3-V4 region sequences from complete genomes of strains in the species Escherichia coli, the genus Escherichia, and the collective genera Escherichia and *Shigella.* We then computed the positional relative entropy of (i) strains within the species E. coli, (ii) non-*coli* strains within the genus Escherichia, and (iii) *Shigella* strains within the total population of Escherichia and *Shigella* (Fig. 1B). Our use of relative entropy therefore identified residue variations that discriminate certain species within the closely related genera Escherichia and *Shigella*.

### Identification of strain- and species-informative rRNA variants.

**FIG 2:**
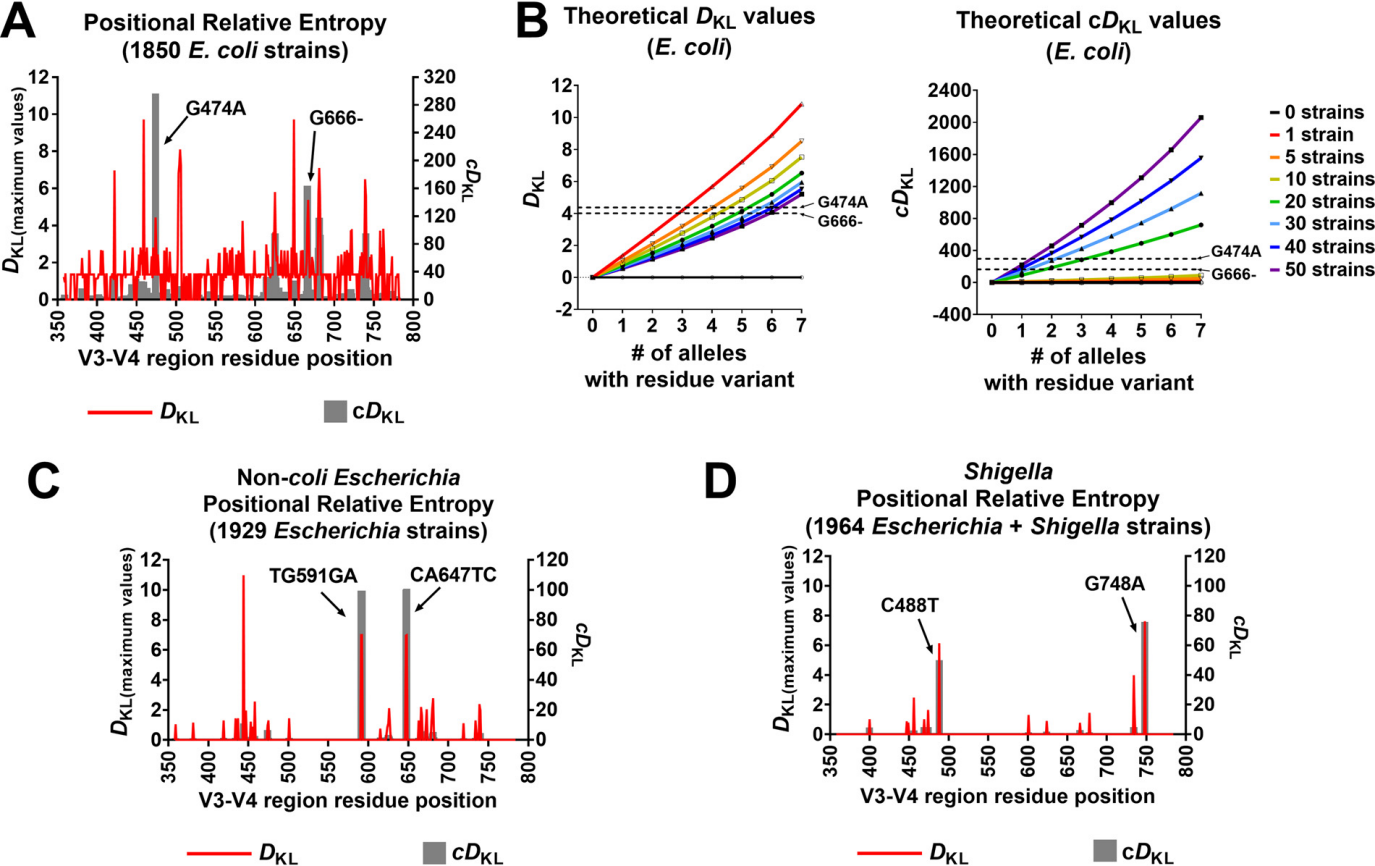
Informative 16S rRNA gene V3-V4 sequence polymorphisms among Escherichia and *Shigella*. Relative entropy was used to identify strain- and species-informative SNPs within Escherichia and *Shigella* V3-V4 sequences across all genomic copies of 16S rRNA genes. (A) A *D*_KL_ peak (red) corresponds to an SNP at that position that is highly correlated with a particular strain among E. coli strains. A peak in a cumulative *D*_KL_ (c*D*_KL_) plot (gray) indicates an SNP that is prevalent across strains and may be specific to E. coli species. For the evaluated E. coli population (1850 strains), 2 positions showed high species c*D*_KL_, corresponding to G474A (c*D*_KL_ = 296.48) and G666- (c*D*_KL_ = 163.86). (B) Theoretical values for *D*_KL_ and c*D*_KL_ were calculated for up to 50 strains in the E. coli population having an SNP in 0 to 7 out of 7 alleles (total of 12,876 sequences in the population). The *D*_KL_ and c*D*_KL_ values of the notable SNPs discussed in (A) are indicated for reference. (C) *D*_KL_ and c*D*_KL_ values were determined for non-*coli* Escherichia strains. Two polymorphisms, TG591GA and CA647TC, had high strain and species values and were found only in *E. albertii* strains. (D) *D*_KL_ and c*D*_KL_ values were calculated to identify *Shigella* strain- and species-SNPs within the large population of Escherichia and *Shigella*. C488T and G748A had high strain and species values and were S. boydii- and S. dysenteriae-informative, respectively.

To illustrate the utility of positional relative entropy (*D*_KL_), we used them to identify V3-V4 residues within Escherichia and *Shigella* that were strain- or species-informative. *D*_KL_ values were determined by comparing the observed residue frequencies at each V3-V4 position per E. coli strain relative to frequencies at that position across 12,876 V3-V4 sequences from 1,850 Escherichia coli strains. We then plotted the maximum *D*_KL_ values as well as the c*D*_KL_ values per position, which aids in visually identifying strain- and species-informative nucleotide deviations, respectively. As such, 2 SNPs exhibited prominent entropy values across multiple E. coli strains (Fig. 2A); an ‘A’ nucleotide at position 474 instead of the consensus ‘G’ (G474A) and an absence of ‘G’ at position 666 (G666-). To assess the relative weights of those values, we compared them to theoretical values of *D*_KL_ and c*D*_KL_ for hypothetical strains harboring 0 to 7 alleles with a specific SNP (Fig. 2B). As the number of alleles with that SNP increases in a genome, so does the *D*_KL_ value, reflecting an increasing uniqueness to that strain in the population. As the number of strains harboring that number of alleles increases in the overall population, the uniqueness decreases, and that SNP becomes less strain-informative. For c*D*_KL_ values, more alleles harboring the SNP increases the information value; however, in this case, the more prevalent an SNP is in the total population, the more species-informative it becomes.

*D*_KL_ and c*D*_KL_ were determined for non-*coli* Escherichia strains, comparing V3-V4 residue frequencies in each strain relative to the total Escherichia population evaluated (13,429 V3-V4 sequences from 1,929 Escherichia strains). Two sets of high *D*_KL_ and c*D*_KL_ values were observed corresponding to TG591GA and CA647TC (Fig. 2C), suggesting they are indicative of a strain and prevalent across strains. These polymorphisms were found exclusively in Escherichia albertii strains (total of 20) in multiple alleles in their genomes and were therefore strongly indicative of this species in a population of Escherichia.

Lastly, *D*_KL_ and c*D*_KL_ were determined for *Shigella* strains, relative to the positional frequencies of residues in the total population of Escherichia and *Shigella* (1,964 strains). This analysis revealed 2 prominent SNPs with high *D*_KL_ and c*D*_KL_ values, C488T and G748A (Fig. 2D). C488T was found in most gene copies among Shigella boydii strains (total of 11) but was also in 1 or 2 gene copies among a few E. coli strains. G748A was exclusively found in Shigella dysenteriae strains (total of 10) and was always present in all 7 copies of the 16S rRNA gene. Also, low *D*_KL_ values for G474A and G666- in the non-E. coli population sets indicates that these SNPs were primarily present in the larger E. coli population. The list of strains and their relative entropy estimations for V3-V4 sequences are available in Data set S1. Overall, positional relative entropy revealed species-informative V3-V4 residues for E. coli, *E. albertii*, S. boydii, and S. dysenteriae.

### Development of a mutant 16S rRNA tracking system.

**FIG 3:**
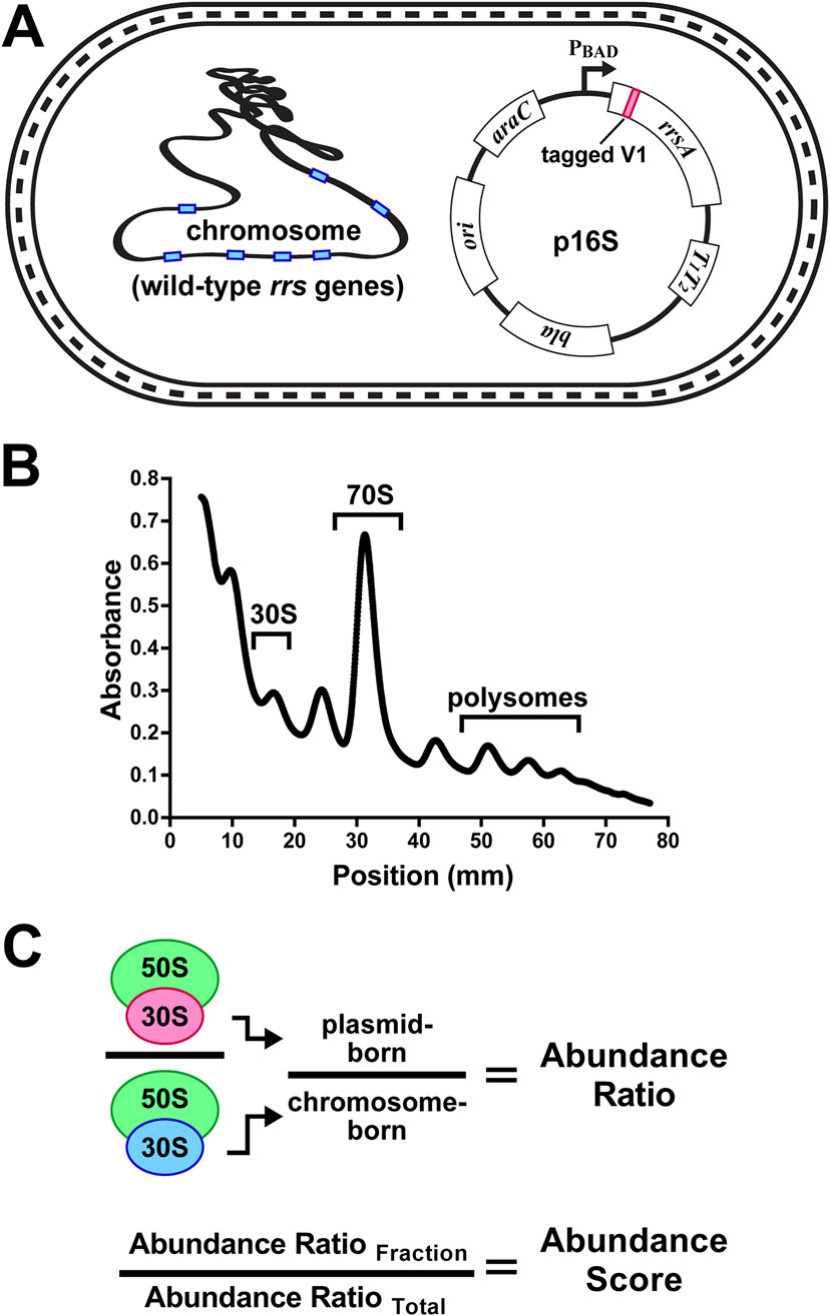
Establishing the performance of 16S rRNA variants. Modified 16S rRNAs were evaluated in an E. coli strain with intact *rrn* operons. (A) The E. coli *rrsA* gene was cloned into a plasmid under the control of a tightly repressed P_BAD_ promoter. The cloned *rrsA* was modified in its variable 1 (V1) region to contain a unique tracking tag sequence that was detectable using RT-qPCR. Other mutations were subsequently introduced in this tagged V1 *rrsA* for abundance evaluations of expressed 16S rRNA. (B) Fractionation of cell lysates using sucrose gradients allowed for isolation of 16S rRNAs in various stages of small subunit assembly and translation. The regions of 30S, 70S, and polysome material collected in this study are indicated. (C) RNA was extracted from gradient fractions and used to establish the abundance ratio of plasmid-born 16S relative to chromosome-born in the same fraction. An abundance score was then calculated by comparing the abundance ratio in a given fraction to that of the unfractionated lysate.

Variable region sequence alterations are not predicted to impact the translation performance of mature ribosomes, but changes to the subunit maturation, ribosome assembly, or selective use in translation may influence evolutionary selection of a particular rRNA sequence. To evaluate the impact of alternative variable region sequences on the performance of 16S rRNAs, we developed a system to track 16S rRNA variants expressed from a plasmid in E. coli having unaltered *rrs* genes in its chromosome (Fig. 3A). This strain lacked *recA* to avoid recombination between plasmid and chromosomal *rrs* alleles. To selectively detect variants among the natural 16S rRNA pool, we tagged the sequence of the plasmid 16S gene such that the expressed rRNA could be selectively detected in cellular fractions among the abundant chromosome-derived 16S rRNAs using reverse transcription and quantitative PCR (RT-qPCR) (Fig. 3B). The candidate position for the sequence tag was helix 6 (H6), which comprises the central portion of variable region 1 (V1 region). This helix generates a spur that extends away from the body of the ribosome (Fig. S1A), and 16S rRNA variants that supported E. coli growth were previously shown to have little sequence conservation in most of this region (32) (Fig. S1B and C). In addition, other groups have used the tip of H6 to host RNA insertions that were used to affinity purify fully functional small subunits (12, 33, 34). Unfortunately, because insertions are flanked by wild-type sequences, we could not selectively monitor plasmid-born variants using a similar approach (Fig. S2A and B). As an alternative, we changed the sequence of H6 while preserving the secondary structure found in the wild-type version (Fig. S2A). We refer to the otherwise unaltered version of this 'V1-tagged' clone as the parental version, into which additional alterations were subsequently introduced.

In prior studies evaluating the functionality of 16S rRNA mutants *in vivo*, the mutant versions were highly expressed (35–40). Having high proportions of experimental 16S rRNAs within ribosome pools was used as an exploitable feature to quantify relative abundance or to generate sufficient material for selective purification of mature particles. However, overexpressing additional rRNA can impart stress if the expressed version is toxic or if it competes for assembly or maturation resources. Our strains harboring plasmids that expressed either untagged 16S rRNA or V1-tagged 16S rRNA became sickened by full plasmid induction and exhibited notably reduced growth rates, whereas a strain containing an empty plasmid grew similarly to the uninduced cultures (Fig. S3). In an effort to discern transcription stress arising from the overproduction of long RNAs from stress caused by a depletion of small subunit resources, we also measured the growth rate of a strain expressing an unrelated non-translated RNA the same length as those from the 16S rRNA plasmids (Fig. S3). These cultures also became sick, but to a lesser extent. We conclude from these experiments that the overexpression of the 16S rRNA caused the sickness and a considerable proportion of this stress may have arisen from resource depletion. Therefore, we chose to evaluate 16S rRNA variants in cells that did not have the plasmids induced.

We observed that the tagged parental 16S rRNAs distributed in sucrose gradients in a manner that mirrored the chromosome-born wild-type versions present in the same cells, which indicated that the plasmid-expressed versions had been processed correctly and incorporated into functional ribosomes (Fig. S4A and B). To establish overall expression levels of each variant and to correct for variable cell harvests and RNA yields between replicate cultures, the tagged 16S rRNA RT-qPCR signals were normalized to those of the untagged chromosomal 16S rRNA signals from each sample. We refer to these values as an 'abundance ratio' (Fig. 3C). In the total lysate RNA preparations presented here, the abundance ratios for the tagged 16S rRNAs varied little among culture replicates (Fig. S4C). Collectively, the chromosomal 16S rRNAs were present at approximately 300 to 500 higher levels than the tagged versions (Fig. S4C). This is an important feature because it allowed for the evaluation of potentially inactive or toxic variants while the abundant wild-type 16S rRNAs supported bulk translation.

We revisited the sickness caused by overexpression of the 16S rRNA by comparing the abundance ratios in a total lysate to the abundance ratios in gradient fractions derived from it. We refer to these values as 'abundance scores' and they represent the performance of a tagged 16S rRNA by comparing its relative abundance in each fraction to its abundance in the total lysate (Fig. 3C). We found that parental 16S rRNA overexpression caused the tagged molecules to heavily over-accumulate in the 30S fraction and to become largely depleted in the 70S and polysome fractions (Fig. S4D). This finding is consistent with the overexpression leading to an inability to generate functional small subunits. We also discovered that parental 16S rRNA in an uninduced culture was present in the 30S fraction at a slightly higher level than in the total lysate, and in a lower level in the 70S and polysome fractions. By this evaluation, 16S rRNA with this version of the V1-tag performed the best out of our cloned collection and it suggests that the alterations to the stem of V1 partially interfered with either maturation, assembly, or initiation. Taken as a limitation of this system, we chose to compare the performance of additional mutants to the performance of the tagged parent using parallel cultures grown and harvested at the same time. The abundance scores for a given mutant were then compared to those of the parent.

### Distribution of toxic 16S rRNA mutants.

**FIG 4:**
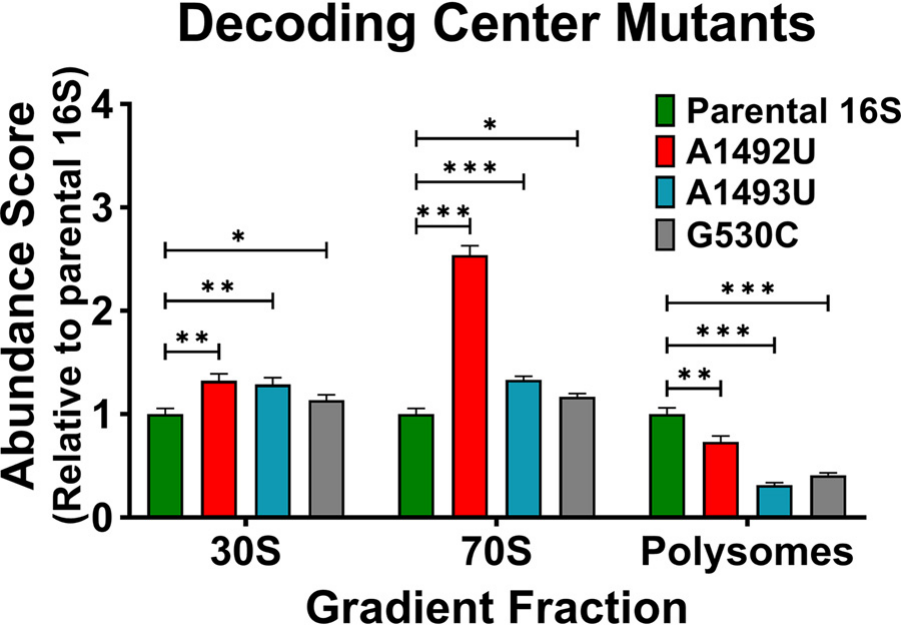
Evaluating decoding center mutants. Abundance scores were determined for plasmid-born 16S rRNA containing separate decoding center mutations A1492U (red), A1493U (blue), and G530C (gray). Scores for mutants were compared to those observed for the parental 16S rRNA (*y* axis) for the 30S, 70S, and polysome lysate fractions. Error bars represent standard deviations for biological replicates (*n* = 3). Comparative statistics represent Student's *t* test results. *P* values <0.05 (*), < 0.01 (**), < 0.001 (***).

To evaluate our test system and to establish comparative abundance scores for 16S rRNAs that are overtly defective, we chose to monitor decoding center mutants. Prior studies have established that alteration of the decoding center residue G530, A1492, or A1493 leads to the formation of small subunits that are dominantly lethal (35, 38, 40, 41), potentially defective in initiation (36), and despite being detected in polysomes at low levels, they were compromised in their ability to translate mRNA (12, 33, 35, 38, 41). We independently introduced G530C, A1492T, and A1493T mutations into the V1-tagged *rrs* gene and calculated abundance scores for the encoded 16S rRNAs present at the 30S, 70S, and polysome gradient positions. Each decoding center mutant was present at a higher abundance than the controls in the 30S and 70S fractions, but at a lower abundance in the polysome pools (Fig. 4).

Although this assay does not establish mechanistic causes for aberrant 16S rRNA distributions, 30S subunits defective in maturation or initiation are expected to over accumulate near the 30S gradient position. Over accumulation in the 70S position may indicate a failure of the ribosomes to properly interact with other translation factors. Whereas a reduced occupancy in the polysome pool may indicate poor initiation, slow translation, increased turnover, or some combination. Therefore, the observed distributions of these decoding center mutants in our assay were consistent with the reported defects at several stages of translation.

### Disparate V3 region sequences affect 16S rRNA abundances in ribosomes.

**FIG 5:**
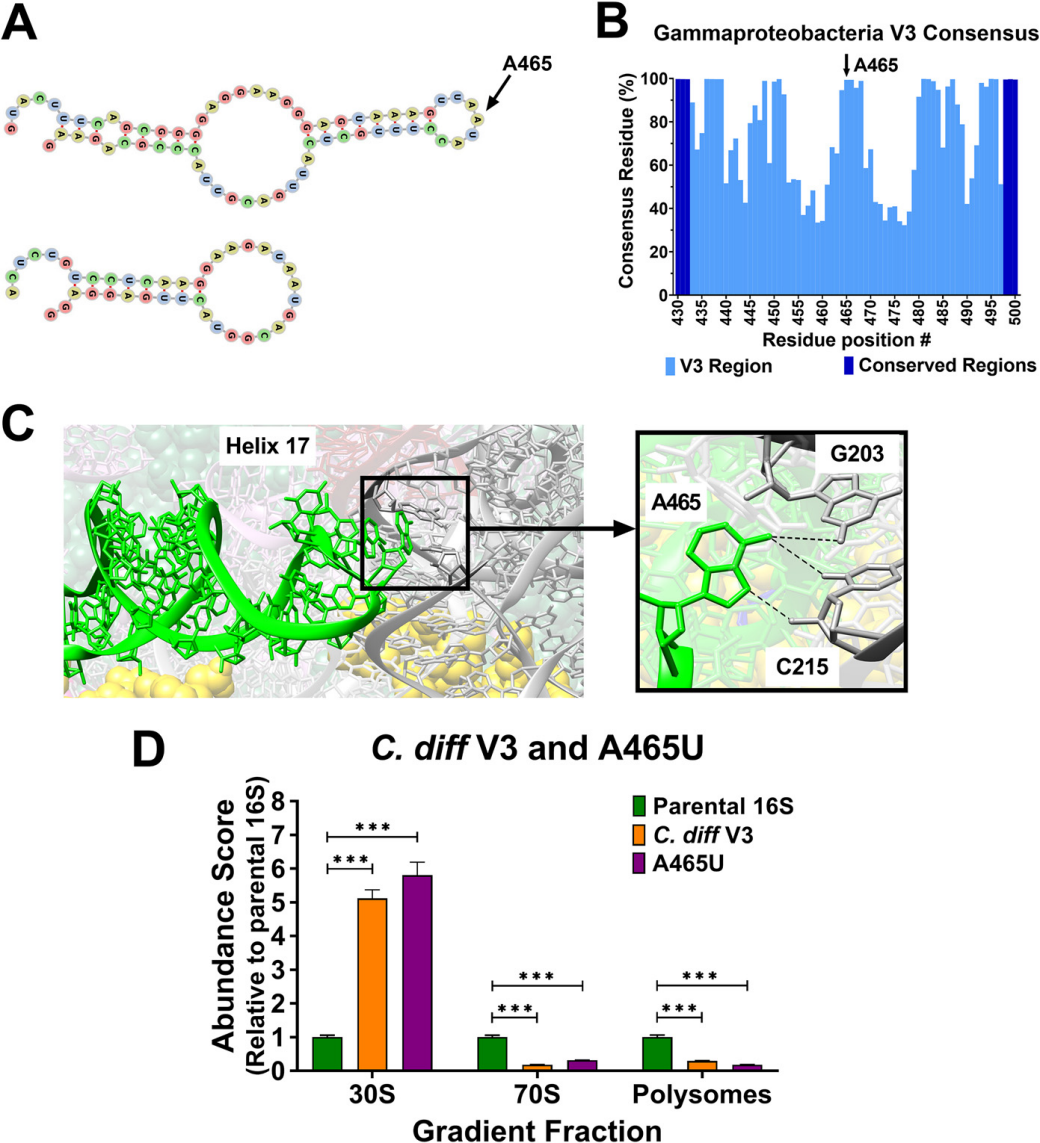
Identification and performance assessment of 16S rRNA with disparate V3 region variants. The central portions of V3 regions are generally not conserved and fall into 2 length categories. (A) Escherichia coli and Clostridioides difficile V3 region secondary structures were computationally predicted using RNAfold (69). The C. difficile V3 encodes a shorter helix and is missing the outer stem-loop. At the tip of the E. coli hairpin is residue A465 (arrow). (B) An analysis of residue consensus in V3 region sequences revealed that A465 is present in over 99% of bacteria in the class *Gammaproteobacteria* (total of 162,325 sequences). (C) In the E. coli ribosome, A465 is located at the tip of helix 17 (lime-green) and forms potential hydrogen bonds with G203 and C215 in the V2 region (dark gray). Yellow spheres are residues of small subunit proteins (image rendered from PDB 4V9D). (D) Abundance scores for E. coli 16S rRNA with *C. diff* V3 (orange) and an A465U transversion (violet) were determined relative to the parent 16S. Error bars represent standard deviations for biological replicates (*n* = 3). Comparative statistics represent Student's *t* test results. *P* value < 0.001(***).

After confirming that our test system provided outputs that reflected the performance of 16S rRNAs, we turned our focus to investigate versions containing alternative V3-V4 regions. 16S variable regions from different bacteria tend to have similar sizes that symmetrically cluster around a mean value (42). One curious exception to this pattern is found in the V3 region encoding helix 17 (H17), the lower stem of which is involved in the assembly of the small subunit in E. coli (43–45). In some species, the central region of V3 lacks approximately 25 residues, while other species in the same genus do not (e.g., members of *Clostridioides*) (Fig. 5A) (42). This observation suggests that the central portion of V3 (encoding the outer stem-loop of H17) is not required and that it can be dispensed in a relatively short evolutionary hop. However, we discovered that the center of this region is highly conserved in the class *Gammaprotobacteria* (which includes E. coli) (Fig. 5B). Moreover, individual 16S alleles within the genomes of comparative reference strains have identical V3 regions (E. coli MG1655 versus Clostridioides difficile 630), suggesting they are not free to drift (Fig. S5). In the mature E. coli ribosome, the tip of H17 interacts with residues of the V2 region (Fig. 5C) (46). Unfortunately, there is no available structure of a C. difficile ribosome for comparison. These striking contrasts in the architecture and conservation of the V3 region raised the question of its importance.

We first tested the performance of an E. coli 16S rRNA harboring the truncated C. difficile V3 region to establish if H17 is required at all. The abundance scores for this variant indicated that these RNAs were defective, and they became trapped at the 30S position, with very little progressing into the 70S or polysome pools (Fig. 5D). Next, we interrogated the tip of H17 by introducing a single SNP (A465U) to disrupt the conservation found in *Gammaproteobacteria*. Remarkably, this variant performed nearly as poorly as the version containing the truncated C. difficile V3 and most of it was retained in the 30S fraction (Fig. 5D). These findings reveal that the tip of H17 plays an important role in some bacteria, but no role in others. Thus, the evolutionary drift rates of the residues within it cannot be equal. These data also highlight the potential for improving taxonomic classifications by focusing on the identities of individual residues that are hallmarks of particular groups of bacteria.

### Escherichia and *Shigella* V3-V4 allele variants influence 16S rRNA performance.

A prior study showed that translation activity in E. coli was compromised when it relied on full-length *Shigella* 16S rRNA as a surrogate (47). Having discovered that Escherichia and *Shigella* species have taxonomically informative nucleotides in variable regions, and that V3 region sequences from species disparate from these 2 genera crippled 16S rRNA performance, we were inspired to determine if the intra-genus species-informative nucleotides we had computationally identified also affected the behavior of E. coli 16S rRNA. Therefore, we characterized versions containing the Escherichia and *Shigella* species-informative V3-V4 variations that were observed in E. coli (G474A and G666-), *E. albertii* (TG591GA and CA647TC), S. boydii (C488T), and S. dysenteriae (G748A).

**FIG 6:**
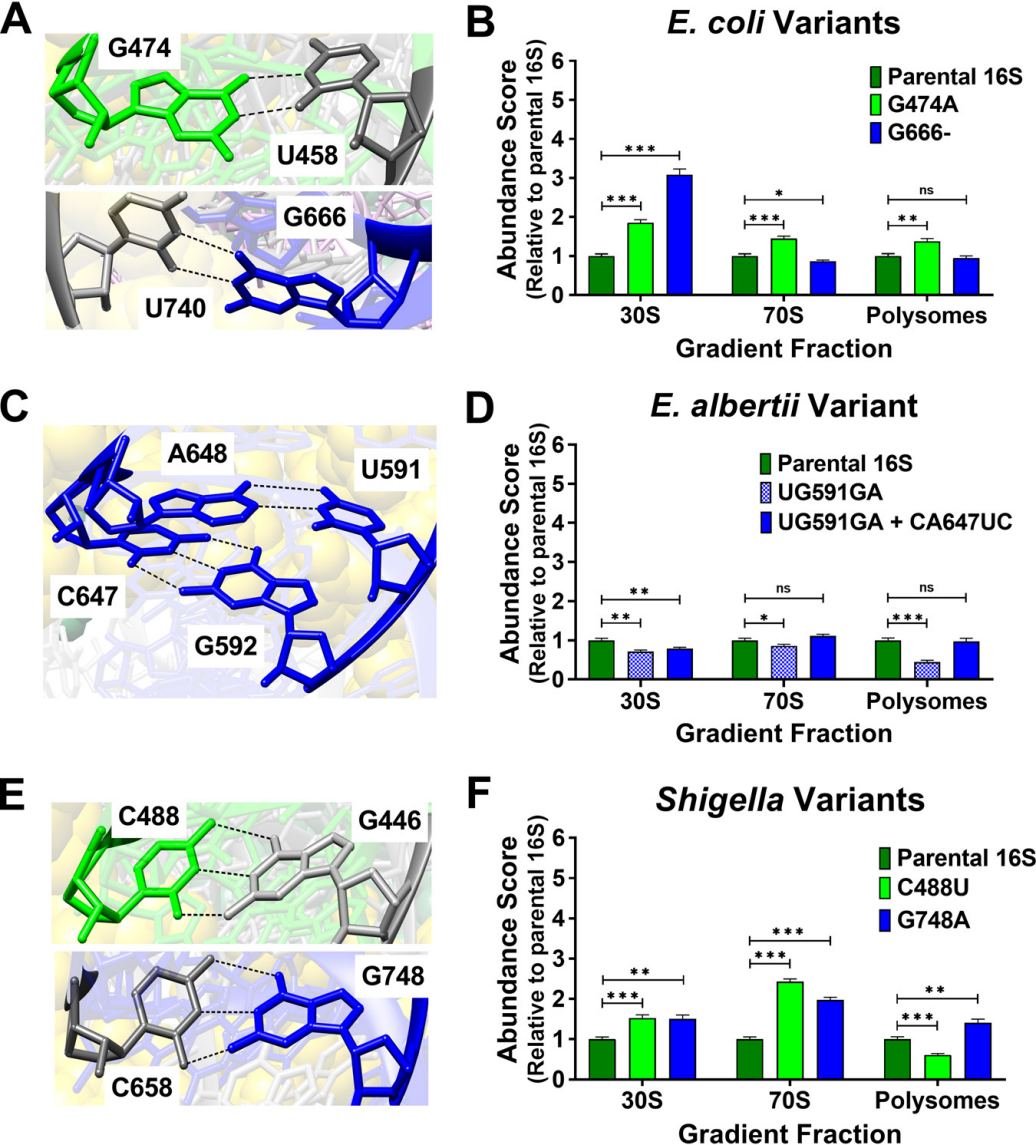
Structure of V3-V4 informative residues in E. coli and abundance scores for Escherichia and *Shigella* species variants. The residue positions for Escherichia and *Shigella* species-informative SNPs were assessed in an of E. coli ribosome structure (PDB 4V9D). Abundance scores were evaluated for E. coli 16S rRNA harboring informative Escherichia and *Shigella* species-informative V3-V4 residues. V3 residues in ribosome structures and abundance scores associated with their mutation are colored lime-green, and those for the V4 region are colored dark blue. Unmutated residues are colored dark gray in structures. (A) G474 hydrogen bonds with U458 and G666 with U740. (B) The abundance scores for 16S rRNAs harboring the species-informative E. coli variations (G474A and G666-) were evaluated *in vivo*. (C) Residues at sites for *E. albertii*-specific polymorphisms (UG591GA and CA647UC) complemented each other. (D) Abundance scores for E. coli 16S rRNAs harboring *E. albertii* V3-V4 variants at 1 or both sites were evaluated. (E) C488 and G748 are positioned to interact with G446 and C658 respectively. (F) Abundance scores for E. coli 16S rRNAs harboring C488U (Shigella boydii SNP) or G748A (Shigella dysenteriae SNP) were evaluated. Error bars represent standard deviations for biological replicates (*n* = 3). Comparative statistics represent Student's *t* test results. *P* values ≥ 0.05 (ns), < 0.05 (*), < 0.01 (**), < 0.001 (***).

Structural studies of E. coli ribosomes showed that G474 is bonded with U458 and G666 is bonded with U740 (Fig. 6A). Curiously, the G474A variant showed abundance scores higher than parental 16S for all fractions, while G666- showed an increase in the 30S score, a modest decrease in 70S, yet normal polysome scores (Fig. 6B). One interpretation of these results is that altering G474 may stabilize ribosomes on messages, while removing G666 slows entry into the ribosome pool, but not performance in polysomes.

The informative *E. albertii* V3-V4 polymorphisms occurred at 2 sites tandemly (TG591GA and CA647TC). In the E. coli ribosome, these transcribed residues exhibit complementary base pairing (Fig. 6C). Observably, these *E. albertii* variations also complement one another. We evaluated 16S rRNA versions with a single site changed (UG591GA) as well as both sites changed (UG591GA and CA647UC). Both polymorphisms exhibited lower 30S scores, however only the single site change exhibited lower scores in 70S and polysome fractions as well (Fig. 6D). Altogether, these results indicate that 16S rRNA abundances among translating ribosomes can be maintained so long as these residues co-vary. Because 4 nucleotide changes are required to convert an E. coli V3-V4 into this *E. albertii* version, and because an intermediate in this conversion performs poorly, these alterations represent a comparatively large evolutionary hurdle, which likely locked these changes into *E. albertii* once they occurred.

The informative S. dysenteriae (G748A) and S. boydii (C488U) polymorphisms would disrupt G-C Watson-Crick bonds found in the E. coli ribosome (Fig. 6E). Interestingly, G748A exhibited increased scores in polysome fractions (Fig. 6F), perhaps indicating more efficient translation initiation or slower egress from the translation pools. In contrast, C488U exhibited lower scores in polysome fractions, indicating that this variant was less capable of entering translation. Importantly, these results show that these *Shigella* species-informative V3-V4 SNPs have a biological impact on 16S rRNA performance, which, as with the *E. albertii* residues, suggests there are indeed functional barriers to their evolutionary drift. Thus, the rates of evolution of variable regions are non-uniform and unpredictable because some organisms may be adapted to particular versions while others are not.

## DISCUSSION

We characterized the performance of 16S rRNA variants with an eye toward establishing whether variable regions contain biologically important residues. This inspection was motivated by the obvious conservation of certain 16S rRNA residues and by the common use of their sequences to establish taxonomy. We observed that residues within variable regions are not necessarily inert and that they can influence the performance of the rRNA they inhabit. Our computational approach revealed residue identities in variable regions that are highly indicative of particular species, even though the overall homologies of those regions render inconclusive species identification. Thus, one approach to refine taxonomy data sets would be to interrogate rRNA sequences that have been grouped into operational taxonomic units for the presence of nucleotide alterations at key positions that reveal a particular species was present in that collection.

Interestingly, we discovered that introducing such species-informative residues into the 16S rRNA of a closely related organism compromised its performance, indicating that the physiology of a bacterium can be fine-tuned to its version of a variable region. Because most bacteria contain multiple *rrn* alleles, conservation of variable regions among them may be indicative of such constraints. These findings prompt a consideration of the molecular mechanisms driving conservation in variable regions. The residues we altered in the V1 and V3-V4 regions are not chemically modified, and they are not known to interact with other factors in E. coli (48, 49). The lowered performance of variants harboring different V1 tags suggests there is some role the native sequence plays in allowing the small subunit to enter the translation pool. For example, perhaps the H6 spur is involved in recruiting late-stage maturation factors. For H17 variations in V3, there is a more pronounced influence on 16S rRNA performance and there is direct structural evidence that this region may help 2 regions of the small subunit interact during assembly in E. coli.

In the Escherichia and *Shigella* V3 sequences, we computationally evaluated that an A465G SNP is present in a single *rrs* allele of only 1 E. coli genome and therefore is neither strain- nor species-informative. However, A465 variations, including U465, were found in 624 other *Gammaproteobacteria* V3 sequences (out of a total of 162,325), none of which were classified as Escherichia or *Shigella*, indicating that a residue other than “A” at position 465 may be indicative of an organism divergent from these 2 genera and that the additional differences in V3 sequence homologies may reflect compensatory adaptations that improve performance. Although it was beyond the scope of this study, similar analyses of different variable regions or other bacterial groups should reveal additional informative residues that indicate the presence of a particular species and a more extensive evolutionary divergence from other members of those groups.

We also identified V3-V4 residues that are informative of non-*coli* species within the genera Escherichia and *Shigella*. E. coli 16S rRNAs harboring the *E. albertii*, S. boydii, or S. dysenteriae species-informative variations exhibited distorted distributions; however, additionally introducing the *E. albertii* 2-site covariation restored performance to that version, which demonstrates the importance of co-variation between certain interacting regions (46, 50). While we suggest that structural changes may be primarily responsible for changes in abundance, we note that covariation in 2D sequence maps may not be sufficient to predict deleterious SNPs. For example, we observed that a 16S rRNA with a Shigella dysenteriae V4 residue exhibited higher abundance scores even though a Watson-Crick bond was disrupted. This study was not designed to identify molecular mechanisms behind aberrant rRNA distributions, but it may be informative in future work to establish residence times of mutants within different ribosome pools to tease apart failures to mature (or to engage other translation factors) from changes in particle stability and lifetime. An additional approach would be to generate strains of bacteria that are forced to rely on a single detrimental variable region to isolate suppressors.

Most bacteria have multiple copies of their rRNA operons (51), so an SNP that reduces rRNA performance may be tolerated if it does not interfere with the activity of the other ribosomes. However, compensatory variations may occur in other parts of that ribosome which may explain, in part, why a strain can have multiple variable region alleles, while a closely related strain may have only 1. In our study, 16S rRNA sequences for certain species could be distinguished by position-specific residue variations that occurred across alleles. As such, we propose that computational tools for establishing relatedness can be improved by incorporating a per residue weighting metric that considers the multi-allele entropy derived from current sequence databases. It would still be necessary to confirm bacterial identity by other metrics (52–54), but computational reevaluations of existing short-read sequence data sets may provide a higher confidence to the presence of particular organisms and offer more detail for evaluating community structures.

## MATERIALS AND METHODS

### Retrieval of 16S gene V1 and V3-V4 sequences and relative entropy analysis.

The NCBI Assembly database was used to download E. coli, Escherichia, and *Shigella* feature table text files (55). Advanced filters for feature tables included “Latest RefSeq,” “Complete genome,” and “RefSeq has annotation”. A Python script was used to extract 16S rRNA gene locations by using keywords “16S ribosomal RNA” and “ribosomal RNA-16S”. Thereafter, using a Python script that incorporated the NCBI efetch tool, the “+” strand sequences of all 16S rRNA gene copies were obtained as a FASTA file. Multiple sequence alignments were carried out using MUSCLE (56). Aliview (57) was then used to remove column gaps observed for all E. coli str. K-12 substr. MG1655 16S sequences across the alignments (NCBI RefSeq# NC_000913.3). E. coli V1 region sequences were extracted based on an E. coli MG1655 reference (8) to determine V1 region nucleotide conservation. Also, the alignment section corresponding to V3-V4 segment (between the S-D-Bact-0341-b-S-17 and S-D-Bact-0785-a-A-21 primer binding sites [58]) was extracted for relative entropy analysis. A Python script incorporating Kullback–Leibler divergence (*D*_KL_) was then executed to determine the V3-V4 positional relative entropy for observing a residue across alleles in a strain compared to the entire population (Equation 1) (31, 59). The output file contained *D*_KL_ per residue position for each strain in the respective populations of study (Data set S1).

(1) $$\mathrm{Positional}\;D_{\mathrm{KL}}=\overset{b}{\underset{t=1}{\sum }}P_{i}\log_{2}\frac{P_{i}}{Q_{i}}$$

Here, *D*_KL_ values are position-specific in the sequence alignment, *P_i_* is the frequency of the observed *t*^th^ residue (A/C/G/T) at the *i*^th^ V3-V4 position across alleles in a strain, *Q_i_* is the reference frequency of that residue at that V3-V4 position across all sequences in the population of study, and *D*_KL_ was used to identify variations specific for E. coli strains in the population of E. coli, non-*coli* Escherichia strains in the population of Escherichia, and *Shigella* strains in the population of Escherichia with *Shigella*. For nucleotides with 0 occurrence, $P_{i}\log_{2}\frac{P_{i}}{Q_{i}}$ was evaluated as 0. A computed value of 0 indicates that there is no positional difference in the residue between the strain and the overall population. A high *D*_KL_ value is indicative of a different residue in multiple alleles of a strain at that position relative to the majority of the population. A cumulative sum of *D*_KL_ (c*D*_KL_) at a position determined if that variation occurred across multiple strains. Designations for strains that contributed to high c*D*_KL_ values were determined if they were a common species.

### Strains and plasmids.

The E. coli *rrsA* locus was PCR amplified from strain BW30270 (an *rph+ fnr+* MG1655 relative, Yale Stock Center CGSC# 7925) initially using a forward primer that annealed in the upstream *hemG* gene to focus the amplification to that *rrs* allele. A second PCR was templated by the initial amplicon that generated a segment lacking the *rrsA* promoters, but retaining the native 5′ and 3′ processing regions, including the 3′ *ileT* and *alaT* genes (corresponding to residues 4,035,375 to 4,037,386 of the NCBI reference sequence NC_000913.3). This amplicon was then enzymatically assembled using NEBuilder (New England Biolabs) with a DNA plasmid fragment such that *rrsA* expression was under the control of a *P_ara_BAD* promoter and a downstream *rrnB* terminator pair derived from plasmid pBAD24N (60). The completed plasmid also contained a p15A replication origin and a *bla* selection gene. V1 sequence tags and other mutations were introduced into the plasmid 16S gene using PCR-based site-directed mutagenesis (61). The experimental host strain was RW401, a Δ*rna::FRT*, Δ*recA::kan*, and Δ*araBAD::cat* derivative of BW30270.

### Experimental culturing, harvesting, and lysis.

Experimental cultures were grown at 37°C in MOPS EZ Rich Defined Medium (Teknova) (62) supplemented with 10 mM sodium bicarbonate, and either 0.2% glycerol (plasmid-born 16S gene expression partially repressed) or 0.2% arabinose (plasmid-born 16S gene expression fully induced). Overnight starter cultures also contained 150 μg/mL ampicillin. On the day of harvests, overnight triplicate cultures were diluted 1:100 in 30 mL of MOPS EZ Rich Defined Medium. Cultures were harvested during exponential phase (absorbance _600 nm_ = 0.2), by promptly transferring cultures from 37°C to 50 mL conical tubes containing 20 mL crushed-ice wash buffer at −80°C (25 mM HEPES Tris, 150 mM sodium chloride, 20 mM magnesium acetate, 1 mM chloramphenicol, and pH = 7.6) which halted translation and aided in keeping ribosomes bound to mRNA. Cells were immediately pelleted (12,500 RCF for 3 min at 2°C). After supernatant removal, cells were resuspended in 450 μL of ice-cold lysis buffer (25 mM HEPES Tris, 100 mM potassium glutamate, 20 mM magnesium acetate, 1 mM chloramphenicol, 14 mM β-mercaptoethanol, 1X Protease Inhibitor Cocktail Set V from Millipore Sigma, and pH = 7.6) and transferred to ice-cold 1.7 mL screw-cap tube containing 100 μL 0.1 mm zirconia/silica beads (BioSpec Products). Lysis was carried out using an MPBio FastPrep-24 5G homogenizer (QuickPrep adapter, 10 m/s, 1 cycle, 30s). Cell debris and beads were pelleted (18,000 RCF for 10 min at 4°C), and the supernatant extracted. Absorbance of the lysate supernatant was determined at 258 nm and normalized to the lowest value among replicates using lysis buffer.

### Cell fractionation.

A total of 10 to 40% sucrose gradients were prepared (solvated in 25 mM HEPES Tris, 100 mM potassium glutamate, 20 mM magnesium acetate, 14 mM β-mercaptoethanol, and 0.5% Tween 20) in polyclear centrifuge tubes (Seton Scientific) using a Gradient Master instrument (BioComp Instruments) the day of culture harvest and kept at 4°C until lysates were prepared. A total 200 μL of normalized lysates were added to the tops of the gradients. Cellular components were separated in the gradients using a Beckman Coulter Optima L-90K ultracentrifuge with a SW41 Ti rotor at 35,000 RPM for 3.5 h at 2°C. The gradients were then fractionated using a BioComp Piston Gradient Fractionator with a Bio-Rad 2110 Fraction Collector while absorbance was measured at 258 nm.

### RNA extraction.

RNA was extracted from 360 μL of each gradient fraction of interest or from a mixture containing 50 μL of total lysate and 310 μL of RNA buffer (10 mM bis-Tris, 0.1 mM EDTA, and pH = 6.5). Samples were mixed with 200 μL 3 M sodium chloride, 3 μL of 10 mg/mL linear polyacrylamide, and 600 μL of acidic phenol-chloroform/isoamyl alcohol (125:24:1, pH = 4.3). The aqueous phase was extracted and washed twice with 300 μL stabilized chloroform. The extracted aqueous phases were then mixed with 700 μL of isopropanol and incubated in ice for at least 30 min followed by precipitate harvesting by cold centrifugation. Without disturbing the pellets, the isopropanol layers were aspirated, and the pellets washed sequentially with 75% and 95% ethanol. The pellets were then dried and resuspended in 50 μL of TURBO DNase reaction mix (~ 2 U/μL) (Invitrogen) with 1 μL Murine RNase Inhibitor (~ 0.8 U/μL) (New England Biolabs). After 45 min incubation at 37°C, 310 μL of RNA buffer was added and the RNA was re-extracted with phenol-chloroform and resuspended in 50 μL of RNA buffer.

### RT-qPCR.

cDNA was generated using the iScript Select cDNA Synthesis Kit (Bio-Rad) following the random primer mix protocol according to the manufacturer’s instructions. Completed cDNA reactions were diluted 10-fold using DNase-free water and used as templates for qPCR using the SsoFast EvaGreen Supermix Kit (Bio-Rad). Three equal volumes of this mix were dispensed per well in a 96-well plate as technical replicates and qPCRs were carried out on a CFX96 Real-Time System (Bio-Rad) programmed for an initial denaturation (95°C for 30 s and cycling at 95°C for 5 s followed by 60°C for 10 s for 40 cycles). Raw qPCR fluorescence data were exported to an online tool for quantification using global fitting (63). The resulting 'seed' values are linearly related to template abundance and were used to calculate plasmid versus chromosomal 16S rRNA abundance ratios and scores. Replicate measurements were averaged and their values and coefficients of variations were used to calculate abundance ratios and associated standard deviations.

### V3 sequence determination of *Gammaproteobacteria* and C. difficile.

A premade multiple sequence alignment of non-redundant *Gammaproteobacteria* 16S rRNA sequences was retrieved from the SILVA database, Ref NR 99 Release 138.1 (64). Jalview (65) was used to obtain the consensus residue percent for the V3 region between residue 433 to 497 using E. coli MG1655 as a reference (8). Separately, 16S gene sequences from C. difficile str. 630 (NCBI RefSeq# NZ_CP010905.2) and E. coli str. K-12 substr. MG1655 (NCBI RefSeq# NC_000913.3) were obtained and aligned using Aliview. Alignment positions corresponding to residues 433 to 497 for the E. coli reference 16S gene were considered as the C. difficile V3 region.

### Structure analysis.

Chimera X was used to view and evaluate an E. coli ribosome (66). All illustrations used PDB 4V9D which contains a ribosome with bound tRNA and was previously used to determine RNA-RNA interactions in E. coli 16S rRNA (67, 68). Hydrogen bonds were illustrated using default parameters, ignoring intra-residue bonds. Putative V1 and V3 region secondary structures were assessed using RNAfold (69). The predicted tagged V1 secondary structure was similar to a biochemically established E. coli V1 secondary structure (68) with the exception of a possible difference in interaction between the residue at position 76 and the residue at position 93 or 94.

### Data availability.

Python scripts used to obtain 16S rRNA gene sequences, the multiple sequence alignment FASTA files for V3-V4 sequences, and Python scripts to determine relative entropy are available on https://github.com/nibose92/Relative-Entropy.git.
