# Supplementary material for: Variable Region Sequences Influence 16S rRNA Performance
Source: Microbiol Spectr. 2023 May 22;11(3):e01252-23. doi: 10.1128/spectrum.01252-23 (PMC10269663; doi:10.1128/spectrum.01252-23)
Supplement: Supplemental file 2 — Figures S1 to S5. Download spectrum.01252-23-s0001.pdf, PDF file, 2.0 MB [file spectrum.01252-23-s0001.pdf]

FIG S1: Structure and sequence analysis of *E. coli* 16S rRNA V1 region. The V1 region of the *E. coli* 16S rRNA was chosen for sequence tagging. **A)** Helix 6 of the 16S rRNA includes the V1 region (red), which protrudes away from the *E. coli* ribosome (PDB: 4V9D) and does not interact with atoms in the 50S proteins (green), 23S rRNA (light gray), 30S proteins (yellow), and most other 16S rRNA (black). The V1 region nucleotides only form hydrogen bonds with other nucleotides in the region (shown in the enlarged image) to maintain the spur structure. **B)** V1 region sequences of 16S rRNA genes and their alleles across *E. coli* strains (12,876 sequences) were aligned and the percent of each nucleotide at each position plotted. **C)** The alignment of V1 region sequences of 16S rRNA gene alleles from *E. coli* str. K-12 substr. MG1655 (NCBI# NC_000913.3) are shown with variable nucleotides colored and in lower case.

A

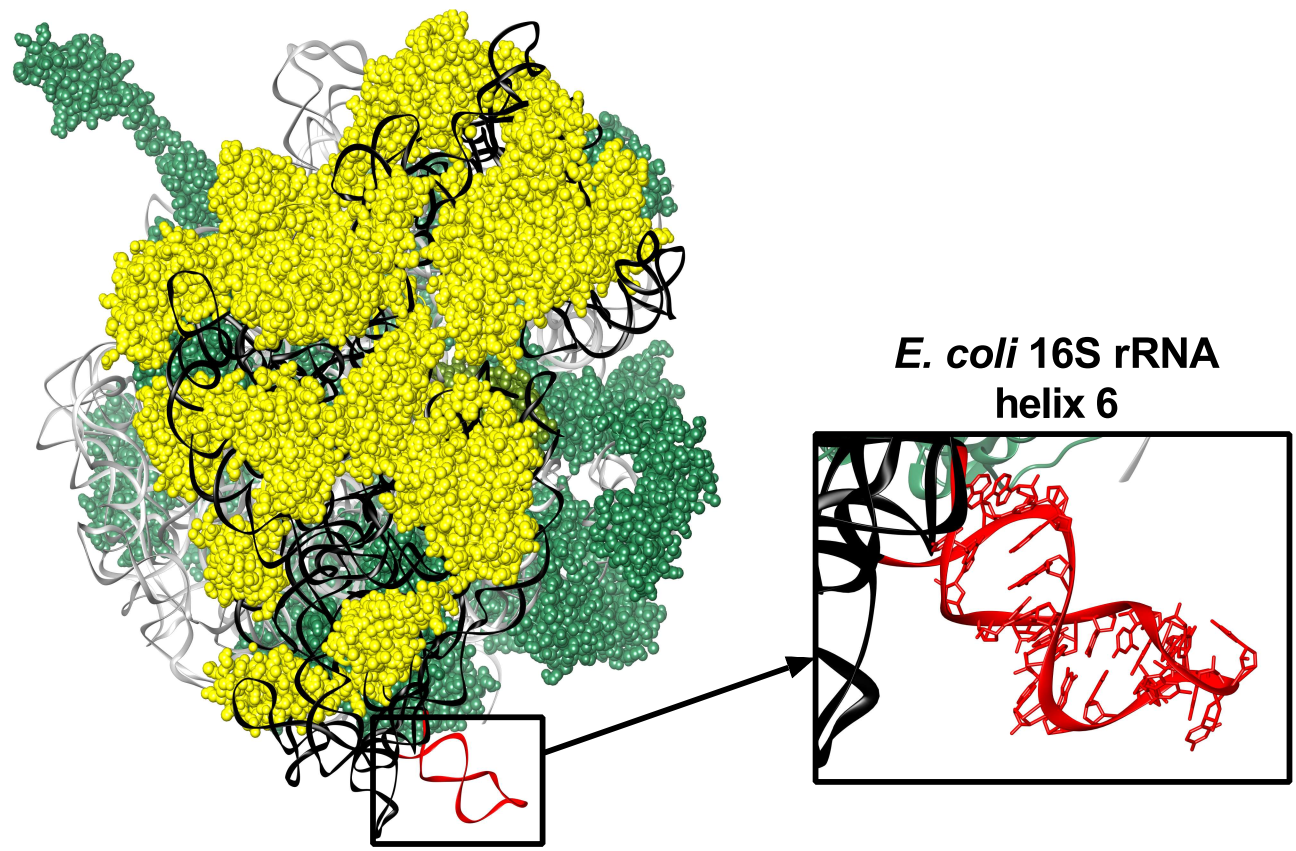

B

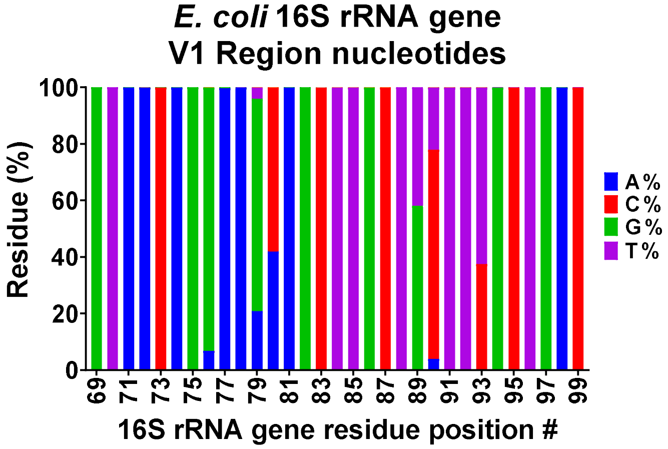

C

***E. coli* str. K-12 substr. MG1655**
16S rRNA gene alleles V1 region

```
rrsA: GTAACAGGAAgaAGCTTGCTtcTTtGCTGAC
rrsB: GTAACAGGAAgaAGCTTGCTtcTTtGCTGAC
rrsC: GTAACAGGAAacAGCTTGCTgtTTcGCTGAC
rrsD: GTAACAGGAAacAGCTTGCTgtTTcGCTGAC
rrsE: GTAACAGGAAgaAGCTTGCTtcTTtGCTGAC
rrsG: GTAACAGGAAgcAGCTTGCTgcTTcGCTGAC
rrsH: GTAACAGGAAgaAGCTTGCTtcTTtGCTGAC
```

FIG S2: Specificity evaluations of qPCR primers for tagged V1 and WT V1: PCR primers that targeted the tagged V1 or WT V1 sequences were evaluated for selectivity using qPCR. **A)** Putative secondary structures of the WT V1 region (derived from *rrsA*) and tagged V1 region bearing various sequence modifications are shown with qPCR primer-binding sites (red for primers targeting tagged V1 and green for primers targeting WT V1). Nucleotides of the tag sequences are in bold and capitalized. The MS2 tagged V1 is the WT V1 with an inserted sequence at the tip of the loop, which was previously used to purify small subunits. The swap-tagged V1 has a portion of the WT stem sequence of helix 6 swapped. The custom tagged V1 sequences contain modifications in the stem sequence that have predicted structures similar to WT V1. **B)** qPCR was performed using purified 16S genes containing the tagged or WT V1 sequences as templates and primers targeting respective tagged sequences or the WT sequence. Templates were normalized to have equal amounts. Specificity was determined by taking the ratio of qPCR seed values for the targeted 16S relative to the non-targeted version. For example, the specificity of the custom tagged V1-1 primers was determined from the ratio of qPCR seeds in reactions with the tagged V1 16S genes (targeted) relative to the WT V1 16S genes (non-targeted). Because the MS2 tagged V1 was an insertion, most of the WT V1 sequence was preserved in the plasmid 16S gene. Therefore, qPCR primers that targeted the WT V1 were "non-specific" because they also targeted the MS2 tagged V1. The specificity of the WT V1 primers was determined from the ratio of qPCR seeds in reactions with the WT V1 16S genes (targeted) relative to each tagged V1 16S genes (non-targeted). qPCR primers targeting custom tagged V1-1 obtained the highest specificity and hence the experimental *rrsA* versions were tagged with this sequence.

A

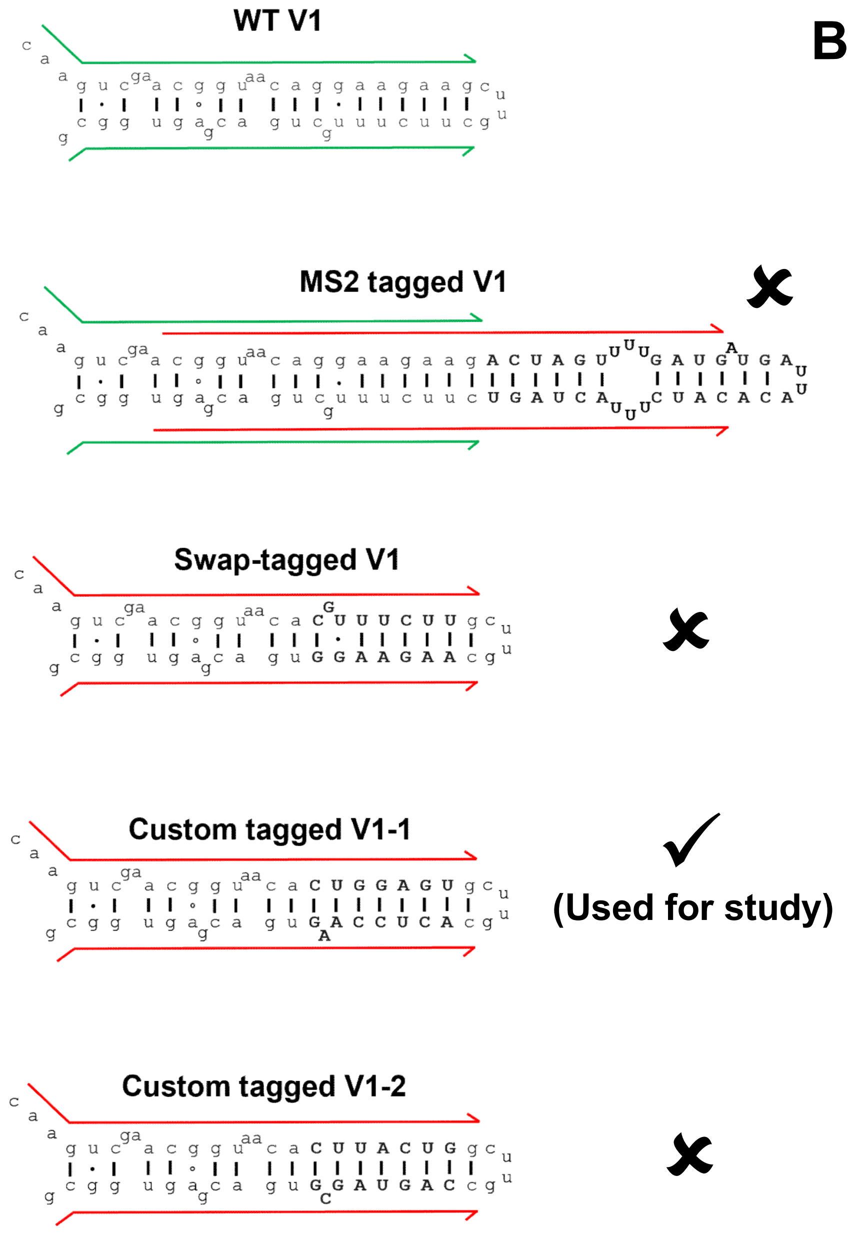

B

Primer	Specificity
MS2 tagged V1	1,402 ± 118
WT V1	Non-specific

Primer	Specificity
Swap-tagged V1	22,389 ± 294
WT V1	334 ± 3

Primer	Specificity
Custom tagged V1-1	259,114 ± 6771
WT V1	418 ± 17

Primer	Specificity
Custom tagged V1-2	119,020 ± 1774
WT V1	580 ± 13

FIG S3: *E. coli* culture growth rates for different plasmid expressions. Doubling times of *E. coli* cultures during exponential phase were determined when plasmids expressed the tagged V1 16S rRNA (Parental 16S) either under uninduced (green) or induced (red) conditions, or when plasmids expressed the untagged WT 16S (orange), a non-translated RNA fragment of the same length derived from the phage Lambda gene encoding gpH (black), or an empty plasmid, pΔ16S, lacking the 16S rRNA gene (gray) under induced condition. Comparative statistics represent student's t-test results. *P* value < 0.001 (***).

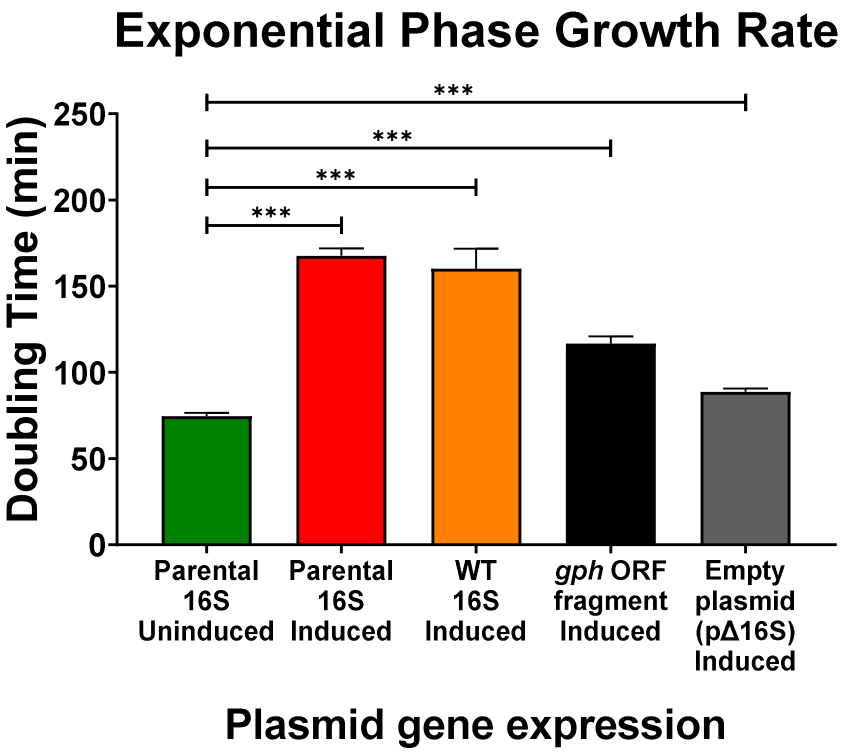

Exponential Phase Growth Rate
Doubling Time (min)
250
200
150
100
50
0
***
***
***
***
Parental 16S Uninduced
Parental 16S Induced
WT 16S Induced
gph ORF fragment Induced
Empty plasmid (pΔ16S) Induced
Plasmid gene expression

FIG S4: Relative abundances of tagged V1 16S for various expression systems. The plasmid-born and chromosome-born 16S rRNA were detected by RT-qPCR in unfractionated lysates as well as gradient fractions 30S, 70S, and polysome pools for uninduced and induced culture growth. **A and B)** RT-qPCR seed values were obtained for uninduced parental V1-tagged and WT chromosome-born 16S cDNAs in each gradient fraction and were normalized to their respective maxima (observed for fraction 6 containing 70S material parental 16S cDNA seed = 6.6×10^{-8}, WT 16S cDNA seed = 2.7×10^{-5}). **C)** The fold excess of the chromosome-born 16S rRNA relative to the plasmid-born 16S rRNA (inverse of the abundance ratio) was determined for unfractionated lysates of cultures containing parental 16S rRNA, or its variants tested in this study, expressed under uninduced conditions. **D)** The abundance ratios for gradient fractions of interest (30S, 70S, polysomes) were divided by their respective ratios for unfractionated cell lysates to obtain abundance scores. Error bars represent standard deviation for biological replicates (n=3).

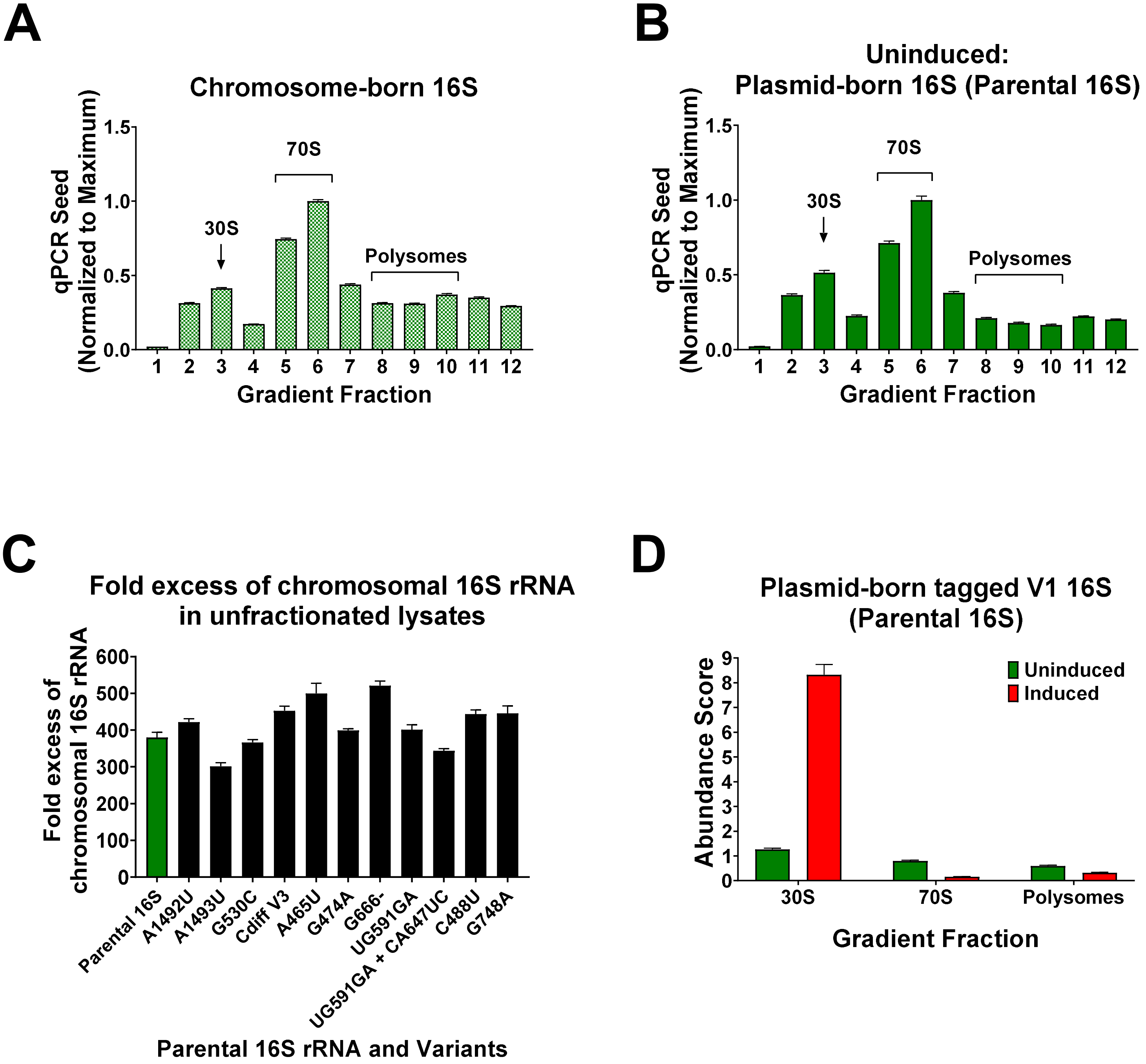
A
Chromosome-born 16S
qPCR Seed
(Normalized to Maximum)
1.5
1.0
0.5
0.0
70S
30S
Polysomes
1 2 3 4 5 6 7 8 9 10 11 12
Gradient Fraction
B
Uninduced:
Plasmid-born 16S (Parental 16S)
qPCR Seed
(Normalized to Maximum)
1.5
1.0
0.5
0.0
70S
30S
Polysomes
1 2 3 4 5 6 7 8 9 10 11 12
Gradient Fraction
C
Fold excess of chromosomal 16S rRNA
in unfractionated lysates
Fold excess of
chromosomal 16S rRNA
600
500
400
300
200
100
0
Parental 16S
A1492U
A1493U
G530C
Cdiff V3
A465U
G474A
G666-
UG591GA
UG591GA + CA647UC
C488U
G748A
Parental 16S rRNA and Variants
D
Plasmid-born tagged V1 16S
(Parental 16S)
Abundance Score
9
8
7
6
5
4
3
2
1
0
Uninduced
Induced
30S
70S
Polysomes
Gradient Fraction

FIG S5: *E. coli* and *C. diff* V3 region alignment. A multiple sequence alignment using MUSCLE within Aliview (57) was performed for 16S gene sequences from *Clostridioides difficile* str. 630 (NCBI# NZ_CP010905.2) and *Escherichia coli* str. K-12 substr. MG1655 (NCBI# NC_000913.3). The V3 region is highlighted in a black rectangle. Residues are colored as: A in green, C in blue, G in black, and T in red. Alleles are identical among genomic copies for the respective strains.

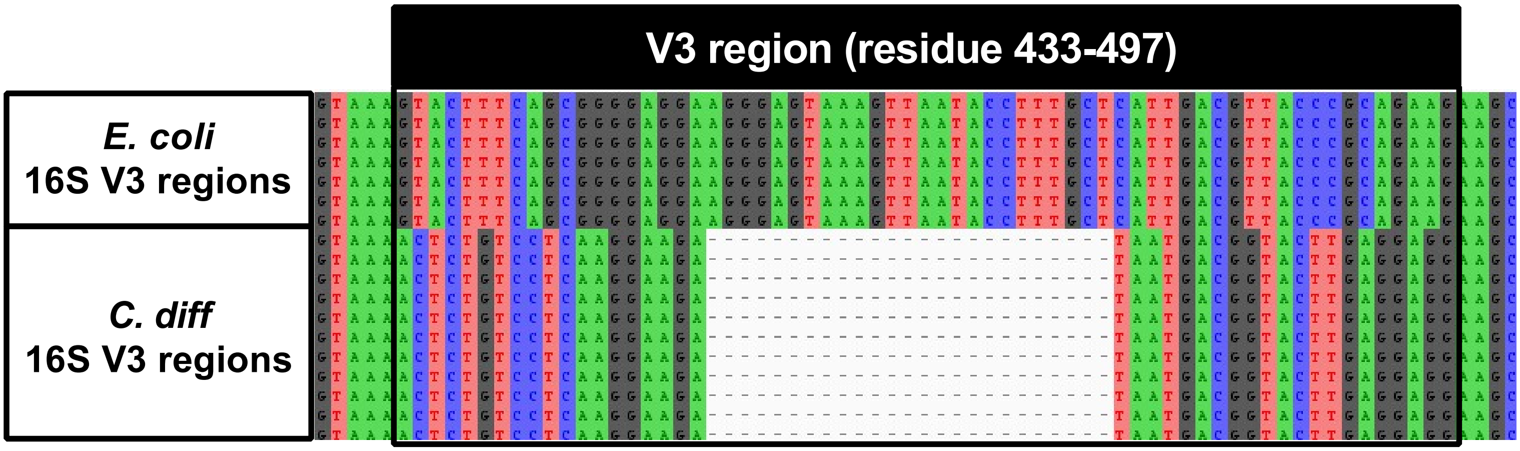
V3 region (residue 433-497)
E. coli
16S V3 regions
C. diff
16S V3 regions

Data Set S1 is a separate Excel file, it contains the list of *Escherichia* and *Shigella* strains retrieved from NCBI RefSeq and relative entropy results of their 16S rRNA gene V3-V4 sequences*.*
